# The Janus Face of Lipids in Human Breast Cancer: How Polyunsaturated Fatty Acids Affect Tumor Cell Hallmarks

**DOI:** 10.1155/2012/712536

**Published:** 2012-07-02

**Authors:** Benoît Chénais, Vincent Blanckaert

**Affiliations:** ^1^EA2160, Mer Molécules Santé, Université du Maine, Avenue Olivier Messiaen, 72085 Le Mans, France; ^2^Département de Biologie, UFR Sciences et Techniques, 72085 Le Mans, France; ^3^Département Génie Biologique, IUT de Laval, 53020 Laval, France

## Abstract

For several years, lipids and especially *n* − 3 and *n* − 6 polyunsaturated fatty acids (PUFAs) receive much attention in human health. Epidemiological studies tend to correlate a PUFA-rich diet with a reduced incidence of cancer, including breast cancer. However, the molecular and cellular mechanisms supporting the effect of PUFAs in breast cancer cells remain relatively unknown. Here, we review some recent progress in understanding the impact that PUFA may have on breast cancer cell proliferation, apoptosis, migration, and invasion. While most of the results obtained with docosahexaenoic acid and/or eicosapentaenoic acid show a decrease of tumor cell proliferation and/or aggressivity, there is some evidence that other lipids, which accumulate in breast cancer tissues, such as arachidonic acid may have opposite effects. Finally, lipids and especially PUFAs appear as potential adjuvants to conventional cancer therapy.

## 1. Introduction

Breast cancer is one of the cancers most frequently observed in industrialized countries, and the one with the highest incidence in women. Epidemiological studies have shown that the rate of breast cancer is 4 to 5 times higher in Western countries that in Japan [1, 2] suggesting that diet, and particularly diet rich in *n* − 3 and *n* − 6 long-chain polyunsaturated fatty acids (PUFAs), may have an influence on tumor emergence [3–6]. Also high dietary intake of fish is associated with a lower incidence of cancers including breast cancer [7–9].

However cohort studies that examined the effect of *n* − 3 PUFAs on breast cancer incidence yielded mixed results, and most of them did not show a significant association between *n* − 3 PUFAs consumption and breast cancer risk [10, 11]. Nevertheless the Women's Intervention Nutrition Study (WINS) provided evidence that a reduction in dietary fat intake to 22% of total energy intake led to a 24% reduction in the recurrence rate of breast cancer [12]. Then, is fat beneficial or not? Probably not in excess, and mainly PUFAs rich fat rather than saturated fat.

Several authors have shown that *n* − 3 PUFAs, namely, docosahexaenoic acid (DHA) and eicosapentaenoic acid (EPA) have demonstrable anticancer properties both *in vitro* and *in vivo*. However, the mechanisms behind the benefits are not clear [13–16]. This paper aims to give a rapid overview of the effect of PUFAs on breast cancer cell proliferation, apoptosis, migration, and invasion.

## 2. Effect of *n* − 3 PUFAs on Breast Cancer Cell Proliferation and Apoptosis

Several studies showed that DHA and EPA together or alone inhibit the growth of breast cancer cell lines [17–19]. Some additional evidence hints that DHA not only acts as an antiproliferative agent by lengthening the cell cycle between the G2/M transition [20], but is also a proapoptotic factor, increasing Bcl-2, procaspase-8, and caspase-3 activity in breast cancer cell lines [21–23]. In addition, DHA has been shown to affect cell proliferation whatever it comes from fish oil or microalgae [24].

Activation of the p44/42 mitogen-activated protein kinase (MAPK) pathway plays a major role in regulating cell growth and survival in breast cancer cells [25] and is protective against apoptosis through phosphorylation of Bad [26]. DHA-induced apoptosis of breast cancer cells was also associated with up-regulation of the transmembrane heparan sulfate proteoglycan syndecan-1 [27]. Moreover increase of syndecan-1 impairs signaling of the MAPK pathway by inhibiting phosphorylation of MEK, Erk, and Bad, that results in apoptosis induction in breast cancer cells [28]. 

Incorporation of *n* − 3 PUFAs in membranes decreased arachidonic acid (AA) content and *n* − 6/*n* − 3 PUFA ratio in the membranes, without modifying the unsaturation index [29]. Consequently, the modification of AA metabolism, especially the inhibition of the production of eicosanoids, may explain in part the antiproliferative and proapoptotic effect of *n* − 3 PUFAs [16, 30]. Whereas low doses of DHA and EPA did not change cell susceptibility to oxidative stress [29], several works report increased lipid peroxidation and ROS production in *n* − 3 PUFA-treated cancer cells associated with growth arrest and apoptosis [31–38]. Some differences between DHA and EPA may be noted as reported in glioblastoma cells where the levels of reactive oxygen species and thiobarbituric acid-reactive substances were significantly higher in DHA-treated cells than in EPA- and AA-treated groups [33]. Glutathione is a key molecule in cellular redox homeostasis, and its reduced form (GSH) content was decreased in DHA-supplemented cells [33, 35]. The activity of cytosolic glutathione peroxidase was decreased in breast cancer cells treated with DHA [37] whereas main antioxidant enzyme activities (i.e., superoxide dismutase and catalase) were increased [33, 37]. The use of various antioxidant molecules was shown to inhibit *n* − 3 PUFA-induced apoptosis suggesting the involvement of lipid peroxidation-derived ROS [31–38]. 

Then PUFAs appeared as proliferation inhibitors and apoptosis inducers in breast cancer cell lines and animal models, at least in part through MAPK and ROS pathways [28, 39].

## 3. Effect of *n* − 3 PUFAs on Lipid Rafts and Signaling in Breast Cancer Cells

The *n* − 3 PUFAs may exert their growth inhibitory effects on cancer cells by altering the plasma membrane composition and associated signaling events [40]. One emerging view is that DHA-containing phospholipids modify the biophysical organization of the plasma membrane which in turn modifies protein activity and cellular function [41–45]. Model membrane studies suggest that the energetically less favorable interaction between cholesterol and PUFA, especially DHA, promotes lateral phase segregation into sterol-poor/PUFA-rich and sterol-rich/saturated fatty acid-rich microdomains [44, 46, 47]. Since lipid rafts are predominantly enriched in saturated fatty acids-containing sphingolipid and cholesterol, the incorporation of PUFA, especially DHA, determines in breast cancer cells a disruption of lipid rafts and a formation of the PUFA-rich/cholesterol-poor nonraft domains [43]. 

The effect of *n* − 3 PUFAs on lipid rafts and their signaling pathways has been studied in several cell types with cancerous origin or not [48]. Particularly, the raft marker caveolin-1 is partially displaced on treatment with DHA and EPA [49, 50]. In the MDA-MB-231 breast cancer cell line, Altenburg and Siddiqui, 2009 showed that *n* − 3 PUFAs exposure resulted in a decreased level of the chemokine receptor CXCR4, which requires intact lipid rafts for signaling [51]. Schley et al, 2007 have shown in the MDA-MB-231 cell line that a combination of EPA and DHA induces a modification in the lipid raft composition including fatty acids, phospholipids, cholesterol, ceramides, and DAG content of membrane rafts [52]. These alterations of lipid content are accompanied by a decrease of EGFR in rafts and an increased whole cells level of phosphorylated EGFR and p38 MAPK [52]. Increased phosphorylation of EGFR and p38 was already reported as a proapoptotic signal in cancer cells [53–55], and especially in the MDA-MB-231 breast cancer cell line [52, 56]. Furthermore DHA induces the upregulation of EGFR tyrosine phosphorylation and the increase of EGFR association with the Sos1 guanine nucleotide protein exchange factor in cancer cell lines including MDA-MB-231 [57]. These data suggest that EGF/Ras/Erk signaling is being disrupted in DHA-treated breast cancer cells by the exclusion of EGFR protein from lipid raft microdomains [52, 57].

Corsetto et al. have recently examined the PUFA incorporation in breast cancer lipid rafts and showed that PUFA are incorporated preferentially in phosphatidylinositol, phosphatidylserine, and phosphatidylcholine that may be relevant to the formation of biologically active metabolites such as prostaglandins, prostacyclins, leukotrienes, resolvines, and protectines [43]. These authors conclude that while EPA may contribute to cell apoptosis mainly through a decrease of AA concentration in lipid raft phospholipids, DHA may change the biophysical properties of lipid rafts decreasing the content of cholesterol and the distribution of key proteins such as EGFR, Src, heterotrimeric G-proteins subunits, or sphyngomyelinase. Indeed DHA decreases the sphingomyelin content in lipid rafts of breast cancer cell lines [43]. This might be due to an activation of sphingomyelinase, leading to the production of ceramide which is well known to be associated with apoptosis and cellular stress [58–61]. Increased activity of the neutral sphingomyelinase in response to EPA and DHA treatment was previously reported in Jurkat leukemic cells [16].

Recently *n* − 3 PUFA-mediated alteration of lipid rafts was linked to oxidative stress modulation. The treatment of rat hepatocytes with EPA increases ethanol-induced oxidative stress via lipid raft aggregation and subsequent phospholipase C*γ* translocation into these microdomains [41]. EPA incorporates preferentially into nonraft membrane region, leading to raft cholesterol increase [41]. In a different model, that is, human endothelial cells, DHA reduced oxidative-stress-induced calcium influx through modification of lipid raft composition [45]. Then what is the effect of *n* − 3 PUFA-mediated remodeling of lipid rafts in breast cancer cells with respect to oxidative stress? 

## 4. PUFA-Induced Inhibition of Cell Migration and Invasiveness

Because metastasis is the leading cause of death from breast cancer, reducing the invasive potential of breast cancer cells is almost as important as destroying in the primary tumor. Recently, we showed that DHA reduces the invasive potential of the MDA-MB-231 breast cancer cell line [21]. Interestingly cholesterol levels in lipid rafts, which are altered by *n* − 3 PUFA [52], are critical for the migration, invasion, and angiogenesis of breast cancer cells [62]. In this study, methyl-*β*-cyclodextrin reduced uPAR and matrix metalloproteinase-9 (MMP-9) colocalization in lipid rafts and inhibited breast carcinoma cell migration and invasion. The decreased expression of uPAR and MMP-9 was reversed by cholesterol supplementation [62]. In addition the DHA-induced reduction of breast cancer cells migration may also be due to inhibition of voltage-gated Na^+^ channels [63, 64]. Indeed DHA inhibits voltage-gated Na^+^ channels (neonatal Na_v_1.5) in a dose-dependent manner, and tetrodoxin, a compound that specifically blocks this type of channels, reduces MDA-MB-231 cell migration at the same level that observed in the presence of DHA [64]. The authors concluded that DHA-induced suppression of cellular migration occurred primarily via down-regulation of voltage-gated Na^+^ channel mRNA and functional protein expression [64]. Moreover voltage-gated Na^+^ channels localization in lipid rafts, such as shown in cardiac cells [65], may be affected by PUFAs [63].

One of the hallmarks for breast cancer metastasis is the over expression of chemokine receptors, which leads to migration of the cancerous cells to surrounding tissues [66–68]. The transmembrane G protein-coupled receptor CXCR4 is the most prominent chemokine receptor expressed in breast cancer cells (but not in normal breast cells) and represent a major factor in breast cancer metastasis due to migration of the cancerous cells through signaling by its unique ligand (i.e., CXCL12) to surrounding tissues [67, 69–71]. Exposure of MDA-MB-231 breast cancer cells to *n* − 3 PUFAs results in decreased surface levels of CXCR4 in a time- and dose-dependent manner [51]. Migration of cells toward the CXCR4 ligand CXCL12 was also significantly reduced on *n* − 3 PUFA treatment [51]. These data suggest that the disruption of required lipid raft domains for CXCR4 signaling and the displacement of CXCR4 from the lipid raft domains are potential mechanisms behind the inhibited migratory response after DHA and EPA treatment [51]. 

## 5. *n* − 6 PUFAs as Promoters of Breast Cancer Cell Proliferation and Invasiveness

Some recent studies focused on the negative effect lipids may have on breast cancer evolution and/or emergence. Both LDL and unsaturated fatty acids have been demonstrated to increase proliferation of estrogen receptor alpha negative (ER^−^) breast cancer cells [72–74] suggesting that higher levels of circulating lipoproteins and free fatty acids, which are common in obesity, metabolic syndrome, and high-fat diets, may themselves promote aggressive characteristics of breast cancer. The observations by Antalis et al. that triple negative breast cancer cell lines (i.e., lacking ER, progesterone receptor, and ERBB2), namely, MDA-MB-231 and MDA-MB-436, had many more lipids droplets as compared to the ER^+^ MCF-7 cell line leads to the investigation of neutral lipid composition and metabolism in these cells [75]. Elevated levels of triacylglycerol and cholesteryl esters were found, with a greater proportion of cholesteryl ester relative to triacylglycerol [75]. Moreover the acylCoA : cholesterol acyltransferase 1 (ACAT1) expression was upregulated as well as the LDL uptake in triple negative cells compared to ER^+^ cells [75]. These data are in agreement with transcriptomic results showing that ACAT1 is more highly expressed in human breast cancer cell lines [76] and human breast tumors [77–80] that were characterized as basal, triple negative, or ER^−^. Interestingly, LDL was shown to stimulate proliferation of ER-cells in a manner dependent from ACAT1 activity [75, 81]. High ACAT1 activity was associated with a higher expression of LDL receptor and both depletion in LDL and ACAT1 inhibition decreased cell migration [81]. This supports the association of lipid accumulation with aggressive behavior in ER-breast cancer cell lines.

Examining fatty acid composition of breast cancer tissues Chang et al. observed higher levels of AA, stearic acid, and DHA in tumoral tissue by comparison with healthy tissue of the same patient [82]. They also observed an increased expression of PPAR*α* in breast cancer tissues [82]. Furthermore, the AA contents of the breast cancer tissues were positively correlated with mammary carcinogenesis [82–84]. Besides these *in vivo* data, AA was found to stimulate the growth rate of three breast cancer cell lines (one triple negative and two triple positive) in correlation with PPAR*α* expression and activity [82]. Invasiveness of breast cancer cells is increased by AA and was recently shown to be dependent of TGF-*β*-activated kinase-1 [85] and ubiquitination of collagen-IV [86]. Treatment of MDA-MB-231 cells with AA also stimulates a signaling pathway dependent of phospholipase-A2-*α*, Src, Erk1/2, and lipoxygenase activities [87]. The mammalian target of rapamycin (mTOR) signaling pathway, which is upregulated in many cancers, was stimulated in breast cancer cultured cells treated with AA [88]. This mTOR pathway seems to be involved in AA-induced proliferation and angiogenesis of breast tumor [88]. In addition, esterification of AA by acyl-CoA synthetase-4 (ACSL4) may play a causal role in the aggressive phenotype of breast cancer cells through the compartmentalization of AA release in mitochondria, a mechanism that serves to drive the specific lipoxygenase metabolism of AA [89].

Like AA, linoleic acid promotes the proliferation and the migration of cancer cells. Linoleic acid induces expression of plasminogen activator inhibitor-1, proliferation, migration, and invasion of breast cancer cells [90, 91]. Linoleic acid also induces focal adhesion kinase, NF-*κ*B activation, matrix metalloproteinase-2 and -9 secretion, and finally cell migration and invasion [92]. By contrast, conjugated linoleic acid (CLA), converted from plant linoleic acid by rumen bacteria, displays the opposite anticancer effect [93]. CLA has been shown to promote breast cancer cell apoptosis through diverse signaling pathways such as estrogen receptor, MAPK, and PI3K/Akt signaling pathways [94–97]. Breast cancer cell invasion is reduced after treatment with CLA, and this may involve PPAR*γ* and E-cadherin/*β*-catenin pathway and/or estrogen receptor and protein phosphatase-2A depending of the cell type [98, 99]. Caveolin-1 expression is also decreased in breast cancer cells treated with CLA [100], leading to lipid raft alteration as observed with *n* − 3 PUFAs.

## 6. Breast Cancer Risk Associated with *n* − 6 and *n* − 3 PUFAs in Human

Animal models have demonstrated that long chain PUFAs have differential effects on mammary tumorigenesis based on double bond position and meta-analyses of mouse mammary tumor models have suggested that *n* − 6 PUFAs, such as linoleic acid and AA have a tumor promoting effect [101–103]. A potential mechanism behind the cancer promoting effects of *n* − 6 PUFAs is through the production of proinflammatory eicosanoids such as prostaglandin E_2_, which promotes angiogenesis and hinders apoptosis. Alternatively, *n* − 3 PUFAs, such as EPA and DHA are the precursor molecules to eicosanoids that are less inflammatory when compared to AA-derived prostanoids [42, 104]. In a case-control study with US women, Bagga et al. showed that total *n* − 6 PUFAs may be contributing to the high risk of breast cancer in the US, and that *n* − 3 PUFAs may have a protective effect [105]. A recent cohort study in China also shows a statistically significant interaction between *n* − 6 PUFA intake, *n* − 3 PUFA intake, and breast cancer risk. Women with lower intake of *n* − 3 PUFA and higher intake of *n* − 6 PUFA had an increased risk for breast cancer compared to women with higher intake of *n* − 3 PUFAs and lower intake of *n* − 6 PUFAs [106]. Accordingly a previous study points out an increased breast cancer risk in Singapore Chinese women belonging to the highest quartile of *n* − 6 fatty acid consumption among subjects who consumed low levels of marine *n* − 3 fatty acids [107]. To conclude, the relative amounts of *n* − 6 PUFA to *n* − 3 PUFAs may be more important for breast cancer risk than individual dietary amounts of these fatty acids.

## 7. Conclusion

High fat diets, obesity, and metabolic syndrome leading to high circulating level of lipoproteins are harmful for more than one aspect, but they may also be aggravating for breast cancer by stimulating tumor proliferation and metastasis. On the contrary PUFAs are generally regarded as safe compounds that are well tolerated and produce few side effects. Their effects on nontumorigenic cells have not been fully elucidated, but some studies suggest that when provided at concentrations that inhibit tumor cell growth, *n* − 3 PUFA exert little or no cytotoxic effects on normal breast cells [17, 18, 108]. Clinical studies are ongoing to show the DHA-improved outcome of chemotherapy in patients with metastatic breast cancer [109, 110]. DHA may also have interesting synergistic effects with other compounds such as with curcumin [111]. Then *n* − 3 PUFA may now be considered as powerful nontoxic adjuvant of canonical anticancer treatments [112], especially for patient suffering from late stage metastatic breast cancer. However, further investigation will be required to determine whether the effects observed in the breast cancer cell lines or animal models will be replicated with primary breast cancer cells, and moreover to see if the *in vitro* observed effects will be translated *in vivo*. Also, it will be important to determine the ratio of *n* − 3 versus *n* − 6 PUFAs needed to be consumed for the beneficiary effect to be achieved.

## References

[1] Holmes MD, Willett WC (2004). Does diet affect breast cancer risk?. *Breast Cancer Research*.

[2] Kinlen LJ (1991). Diet and breast cancer. *British Medical Bulletin*.

[3] Blanckaert VD, Schelling ME, Elstad CA, Meadows GG (1993). Differential growth factor production, secretion, and response by high and low metastatic variants of B16BL6 melanoma. *Cancer Research*.

[4] Greenwald P (2001). Clinical trials of breast and prostate cancer prevention. *Journal of Nutrition*.

[5] Molokhia EA, Perkins A (2008). Preventing Cancer. *Primary Care*.

[6] Berquin IM, Edwards IJ, Chen YQ (2008). Multi-targeted therapy of cancer by omega-3 fatty acids. *Cancer Letters*.

[7] Hursting SD, Thornquist M, Henderson MM (1990). Types of dietary fat and the incidence of cancer at five sites. *Preventive Medicine*.

[8] Kaizer L, Boyd NF, Kriukov V, Tritchler D (1989). Fish consumption and breast cancer risk: an ecological study. *Nutrition and Cancer*.

[9] Sasaki S, Horacsek M, Kesteloot H (1993). An ecological study of the relationship between dietary fat intake and breast cancer mortality. *Preventive Medicine*.

[10] Holmes MD, Liu S, Hankinson SE, Colditz GA, Hunter DJ, Willett WC (2004). Dietary carbohydrates, fiber, and breast cancer risk. *American Journal of Epidemiology*.

[11] MacLean CH, Newberry SJ, Mojica WA (2006). Effects of omega-3 fatty acids on cancer risk: a systematic review. *The Journal of the American Medical Association*.

[12] Chlebowski RT, Blackburn GL, Thomson CA (2006). Dietary fat reduction and breast cancer outcome: interim efficacy results from the women’s intervention nutrition study. *Journal of the National Cancer Institute*.

[13] Connolly JM, Gilhooly EM, Rose DP (1999). Effects of reduced dietary linoleic acid intake, alone or combined with an algal source of docosahexaenoic acid, on MDA-MB-231 breast cancer cell growth and apoptosis in nude mice. *Nutrition and Cancer*.

[14] Noguchi M, Taniya T, Kumaki T (1997). Dietary fat and breast cancer: a controversial issue. *Breast Cancer*.

[15] Rose DP, Connolly JM (1999). Omega-3 fatty acids as cancer chemopreventive agents. *Pharmacology and Therapeutics*.

[16] Wu M, Harvey KA, Ruzmetov N (2005). Omega-3 polyunsaturated fatty acids attenuate breast cancer growth through activation of a neutral sphingomyelinase-mediated pathway. *International Journal of Cancer*.

[17] Chajes V, Sattler W, Stranzl A, Kostner GM (1995). Influence of *n* − 3 fatty acids on the growth of human breast cancer cells in vitro: relationship to peroxides and vitamin-E. *Breast Cancer Research and Treatment*.

[18] Grammatikos SI, Subbaiah PV, Victor TA, Miller WM (1994). *n* − 3 and *n* − 6 fatty acid processing and growth effects in neoplastic and non-cancerous human mammary epithelial cell lines. *British Journal of Cancer*.

[19] Schley PD, Jijon HB, Robinson LE, Field CJ (2005). Mechanisms of omega-3 fatty acid-induced growth inhibition in MDA-MB-231 human breast cancer cells. *Breast Cancer Research and Treatment*.

[20] Barascu A, Besson P, Le Floch O, Bougnoux P, Jourdan ML (2006). CDK1-cyclin B1 mediates the inhibition of proliferation induced by omega-3 fatty acids in MDA-MB-231 breast cancer cells. *International Journal of Biochemistry and Cell Biology*.

[21] Blanckaert V, Ulmann L, Mimouni V, Antol J, Brancquart L, Chénais B (2010). Docosahexaenoic acid intake decreases proliferation, increases apoptosis and decreases the invasive potential of the human breast carcinoma cell line MDA-MB-231. *International Journal of Oncology*.

[22] Chamras H, Ardashian A, Heber D, Glaspy JA (2002). Fatty acid modulation of MCF-7 human breast cancer cell proliferation, apoptosis and differentiation. *Journal of Nutritional Biochemistry*.

[23] Corsetto PA, Montorfano G, Zava S, Jovenitti IE, Cremona A, Rizzo AM (2011). Effects of *n* − 3 PUFAs on breast cancer cells through their incorporation in plasma membrane. *Lipids in Health and Disease*.

[24] Judé S, Roger S, Martel E (2006). Dietary long-chain omega-3 fatty acids of marine origin: a comparison of their protective effects on coronary heart disease and breast cancers. *Progress in Biophysics and Molecular Biology*.

[25] Santen RJ, Song RX, McPherson R (2002). The role of mitogen-activated protein (MAP) kinase in breast cancer. *Journal of Steroid Biochemistry and Molecular Biology*.

[26] McCubrey JA, Steelman LS, Chappell WH (2007). Roles of the Raf/MEK/ERK pathway in cell growth, malignant transformation and drug resistance. *Biochimica et Biophysica Acta*.

[27] Sun H, Berquin IM, Owens RT, O’Flaherty JT, Edwards IJ (2008). Peroxisome proliferator-activated receptor *γ*-mediated up-regulation of syndecan-1 by *n* − 3 fatty acids promotes apoptosis of human breast cancer cells. *Cancer Research*.

[28] Sun H, Hu Y, Gu Z, Owens RT, Chen YQ, Edwards IJ (2011). Omega-3 fatty acids induce apoptosis in human breast cancer cells and mouse mammary tissue through syndecan-1 inhibition of the MEK-Erk pathway. *Carcinogenesis*.

[29] Calviello G, Palozza P, Franceschelli P, Bartoli GM (1997). Low-dose eicosapentaenoic or docosahexaenoic acid administration modifies fatty acid composition and does not affect susceptibility to oxidative stress in rat erythrocytes and tissues. *Lipids*.

[30] Hammamieh R, Sumaida D, Zhang XY, Das R, Jett M (2007). Control of the growth of human breast cancer cells in culture by manipulation of arachidonate metabolism. *BMC Cancer*.

[31] Kang KS, Wang P, Yamabe N, Fukui M, Jay T, Zhu BT (2010). Docosahexaenoic acid induces apoptosis in MCF-7 cells in vitro and in vivo via reactive oxygen species formation and caspase 8 activation. *PLoS ONE*.

[32] Kikawa KD, Herrick JS, Tateo RE, Mouradian M, Tay JS, Pardini RS (2010). Induced oxidative stress and cell death in the A549 lung adenocarcinoma cell line by ionizing radiation is enhanced by supplementation with docosahexaenoic acid. *Nutrition and Cancer*.

[33] Leonardi F, Attorri L, Di Benedetto R (2005). Effect of arachidonic, eicosapentaenoic and docosahexaenoic acids on the oxidative status of C6 glioma cells. *Free Radical Research*.

[34] Lindskog M, Gleissman H, Ponthan F, Castro J, Kogner P, Johnsen JI (2006). Neuroblastoma cell death in response to docosahexaenoic acid: sensitization to chemotherapy and arsenic-induced oxidative stress. *International Journal of Cancer*.

[35] Merendino N, Loppi B, D’Aquino M (2005). Docosahexaenoic acid induces apoptosis in the human PaCa-44 pancreatic cancer cell line by active reduced glutathione extrusion and lipid peroxidation. *Nutrition and Cancer*.

[36] Saw CLL, Huang Y, Kong AN (2010). Synergistic anti-inflammatory effects of low doses of curcumin in combination with polyunsaturated fatty acids: docosahexaenoic acid or eicosapentaenoic acid. *Biochemical Pharmacology*.

[37] Vibet S, Goupille C, Bougnoux P, Steghens JP, Goré J, Mahéo K (2008). Sensitization by docosahexaenoic acid (DHA) of breast cancer cells to anthracyclines through loss of glutathione peroxidase (GPx1) response. *Free Radical Biology and Medicine*.

[38] Siddiqui RA, Harvey K, Stillwell W (2008). Anticancer properties of oxidation products of docosahexaenoic acid. *Chemistry and Physics of Lipids*.

[39] Sun H, Hu Y, Gu Z (2011). Endogenous synthesis of n-3 polyunsaturated fatty acids in Fat-1 mice is associated with increased mammary gland and liver syndecan-1. *PLoS ONE*.

[40] Chapkin RS, Mcmurray DN, Davidson LA, Patil BS, Fan YY, Lupton JR (2008). Bioactive dietary long-chain fatty acids: emerging mechanisms of action. *British Journal of Nutrition*.

[41] Aliche-Djoudi F, Podechard N, Chevanne M (2011). Physical and chemical modulation of lipid rafts by a dietary *n* − 3 polyunsaturated fatty acid increases ethanol-induced oxidative stress. *Free Radical Biology and Medicine*.

[42] Calder PC (2011). Fatty acids and inflammation: the cutting edge between food and pharma. *European Journal of Pharmacology*.

[43] Corsetto PA, Cremona A, Montorfano G Chemical-physical changes in cell membrane microdomains of breast cancer cells after omega-3 PUFA incorporation.

[44] Shaikh SR, LoCascio DS, Soni SP, Wassall SR, Stillwell W (2009). Oleic- and docosahexaenoic acid-containing phosphatidylethanolamines differentially phase separate from sphingomyelin. *Biochimica et Biophysica Acta*.

[45] Ye S, Tan L, Ma J, Shi Q, Li J (2010). Polyunsaturated docosahexaenoic acid suppresses oxidative stress induced endothelial cell calcium influx by altering lipid composition in membrane caveolar rafts. *Prostaglandins Leukotrienes and Essential Fatty Acids*.

[46] Wassall SR, Stillwell W (2008). Docosahexaenoic acid domains: the ultimate non-raft membrane domain. *Chemistry and Physics of Lipids*.

[47] Wassall SR, Stillwell W (2009). Polyunsaturated fatty acid-cholesterol interactions: domain formation in membranes. *Biochimica et Biophysica Acta*.

[48] Siddiqui RA, Harvey KA, Zaloga GP, Stillwell W (2007). Modulation of lipid rafts by omega-3 fatty acids in inflammation and cancer: implications for use of lipids during nutrition support. *Nutrition in Clinical Practice*.

[49] Li Q, Zhang Q, Wang M (2007). Docosahexaenoic acid affects endothelial nitric oxide synthase in caveolae. *Archives of Biochemistry and Biophysics*.

[50] Li Q, Zhang Q, Wang M, Zhao S, Xu G, Li J (2008). n-3 polyunsaturated fatty acids prevent disruption of epithelial barrier function induced by proinflammatory cytokines. *Molecular Immunology*.

[51] Altenburg JD, Siddiqui RA (2009). Omega-3 polyunsaturated fatty acids down-modulate CXCR4 expression and function in MDA-MB-231 breast cancer cells. *Molecular Cancer Research*.

[52] Schley PD, Brindley DN, Field CJ (2007). (*n* − 3) PUFA alter raft lipid composition and decrease epidermal growth factor receptor levels in lipid rafts of human breast cancer cells. *Journal of Nutrition*.

[53] Im E, Martinez JD (2004). Ursodeoxycholic acid (UDCA) can inhibit deoxycholic acid (DCA)-induced apoptosis via modulation of EGFR/Raf-1/ERK signaling in human colon cancer cells. *Journal of Nutrition*.

[54] Qiu L, Zhou C, Sun Y (2006). Paclitaxel and ceramide synergistically induce cell death with transient activation of EGFR and ERK pathway in pancreatic cancer cells. *Oncology Reports*.

[55] Squires MS, Hudson EA, Howells L (2003). Relevance of mitogen activated protein kinase (MAPK) and phosphotidylinositol-3-kinase/protein kinase B (PI3K/PKB) pathways to induction of apoptosis by curcumin in breast cells. *Biochemical Pharmacology*.

[56] Cuadrado A, García-Fernández LF, González L (2003). Aplidin induces apoptosis in human cancer cells via glutathione depletion and sustained activation of the epidermal growth factor receptor, Src, JNK, and p38 MAPK. *The Journal of Biological Chemistry*.

[57] Rogers KR, Kikawa KD, Mouradian M (2010). Docosahexaenoic acid alters epidermal growth factor receptor-related signaling by disrupting its lipid raft association. *Carcinogenesis*.

[58] Dimanche-Boitrel MT, Rebillard A, Gulbins E (2011). Ceramide in chemotherapy of tumors. *Recent Patents on Anti-Cancer Drug Discovery*.

[59] Furuya H, Shimizu Y, Kawamori T (2011). Sphingolipids in cancer. *Cancer and Metastasis Reviews*.

[60] Lafont E, Kitatani K, Okazaki T, Segui B (2011). Regulation of death and growth signals at the plasma membrane by sphingomyelin synthesis: implications for hematological malignancies. *Recent Patents on Anti-Cancer Drug Discovery*.

[61] Nikolova-Karakashian MN, Rozenova KA (2010). Ceramide in stress response. *Advances in Experimental Medicine and Biology*.

[62] Raghu H, Sodadasu PK, Malla RR, Gondi CS, Estes N, Rao JS (2010). Localization of uPAR and MMP-9 in lipid rafts is critical for migration, invasion and angiogenesis in human breast cancer cells. *BMC Cancer*.

[63] Gillet L, Roger S, Bougnoux P, Le Guennec JY, Besson P (2011). Beneficial effects of omega-3 long-chain fatty acids in breast cancer and cardiovascular diseases: voltage-gated sodium channels as a common feature?. *Biochimie*.

[64] Isbilen B, Fraser SP, Djamgoz MBA (2006). Docosahexaenoic acid (omega-3) blocks voltage-gated sodium channel activity and migration of MDA-MB-231 human breast cancer cells. *International Journal of Biochemistry and Cell Biology*.

[65] Maguy A, Hebert TE, Nattel S (2006). Involvement of lipid rafts and caveolae in cardiac ion channel function. *Cardiovascular Research*.

[66] Hiller D, Chu QD (2011). CXCR4 and axillary lymph nodes: review of a potential biomarker for breast cancer metastasis. *International Journal of Breast Cancer*.

[67] Dewan MZ, Ahmed S, Iwasaki Y, Ohba K, Toi M, Yamamoto N (2006). Stromal cell-derived factor-1 and CXCR4 receptor interaction in tumor growth and metastasis of breast cancer. *Biomedicine and Pharmacotherapy*.

[68] Müller A, Homey B, Soto H (2001). Involvement of chemokine receptors in breast cancer metastasis. *Nature*.

[69] Chen X, Beutler JA, McCloud TG (2003). Tannic acid is an inhibitor of CXCL12 (SDF-1*α*)/CXCR4 with antiangiogenic activity. *Clinical Cancer Research*.

[70] Fernandis AZ, Prasad A, Band H, Klösel R, Ganju RK (2004). Regulation of CXCR4-mediated chemotaxis and chemoinvasion of breast cancer cells. *Oncogene*.

[71] Kang H, Watkins G, Parr C, Douglas-Jones A, Mansel RE, Jiang WG (2005). Stromal cell derived factor-1: its influence on invasiveness and migration of breast cancer cells in vitro, and its association with prognosis and survival in human breast cancer. *Breast Cancer Research*.

[72] Chajès V, Mahon M, Kostner GM (1996). Influence of LDL oxidation on the proliferation of human breast cancer cells. *Free Radical Biology and Medicine*.

[73] Hardy S, El-Assaad W, Przybytkowski E, Joly E, Prentki M, Langelier Y (2003). Saturated fatty acid-induced apoptosis in MDA-MB-231 breast cancer cells. A role for cardiolipin. *The Journal of Biological Chemistry*.

[74] Rotheneder M, Kostner GM (1989). Effects of low- and high-density lipoproteins on the proliferation of human breast cancer cells in vitro: differences between hormone-dependent and hormone-independent cell lines. *International Journal of Cancer*.

[75] Antalis CJ, Arnold T, Rasool T, Lee B, Buhman KK, Siddiqui RA (2010). High ACAT1 expression in estrogen receptor negative basal-like breast cancer cells is associated with LDL-induced proliferation. *Breast Cancer Research and Treatment*.

[76] Neve RM, Chin K, Fridlyand J (2006). A collection of breast cancer cell lines for the study of functionally distinct cancer subtypes. *Cancer Cell*.

[77] Farmer P, Bonnefoi H, Becette V (2005). Identification of molecular apocrine breast tumours by microarray analysis. *Oncogene*.

[78] Richardson AL, Wang ZC, de Nicolo A (2006). X chromosomal abnormalities in basal-like human breast cancer. *Cancer Cell*.

[79] van de Vijver MJ, He YD, van’T Veer LJ (2002). A gene-expression signature as a predictor of survival in breast cancer. *The New England Journal of Medicine*.

[80] van’t Veer LJ, Dai H, van de Vijver MJ (2002). Gene expression profiling predicts clinical outcome of breast cancer. *Nature*.

[81] Antalis CJ, Uchida A, Buhman KK, Siddiqui RA (2011). Migration of MDA-MB-231 breast cancer cells depends on the availability of exogenous lipids and cholesterol esterification. *Clinical and Experimental Metastasis*.

[82] Chang NW, Wu CT, R CD, Yeh CY, Lin C High levels of arachidonic acid and peroxisome proliferator-activated receptor-alpha in breast cancer tissues are associated with promoting cancer cell proliferation.

[83] Hietanen E, Punnonen K, Punnonen R, Auvinen O (1986). Fatty acid composition of phospholipids and neutral lipids and lipid peroxidation in human breast cancer and lipoma tissue. *Carcinogenesis*.

[84] Maillard V, Bougnoux P, Ferrari P (2002). *n* − 3 and *n* − 6 fatty acids in breast adipose tissue and relative risk of breast cancer in a case-control study in tours, France. *International Journal of Cancer*.

[85] Ray DM, Myers PH, Painter JT, Hoenerhoff MJ, Olden K, Roberts JD Inhibition of transforming growth factor-beta-activated kinase-1 blocks cancer cell adhesion, invasion, and metastasis.

[86] Ray DM, Rogers BA, Sunman JA, Akiyama SK, Olden K, Roberts JD (2010). Lysine 63-linked ubiquitination is important for arachidonic acid-induced cellular adhesion and migration. *Biochemistry and Cell Biology*.

[87] Villegas-Comonfort S, Serna-Marquez N, Galindo-Hernandez O, Navarro-Tito N, Salazar EP Arachidonic acid induces an increase of beta-1,4-galactosyltransferase I expression in MDA-MB-231 breast cancer cells.

[88] Wen ZH, Su YC, Lai PL Critical role of arachidonic acid-activated mTOR signaling in breast carcinogenesis andangiogenesis.

[89] Maloberti PM, Duarte AB, Orlando UD (2010). Functional interaction between acyl-coa synthetase 4, lipooxygenases and cyclooxygenase-2 in the aggressive phenotype of breast cancer cells. *PLoS ONE*.

[90] Byon CH, Hardy RW, Ren C (2009). Free fatty acids enhance breast cancer cell migration through plasminogen activator inhibitor-1 and SMAD4. *Laboratory Investigation*.

[91] Reyes N, Reyes I, Tiwari R, Geliebter J (2004). Effect of linoleic acid on proliferation and gene expression in the breast cancer cell line T47D. *Cancer Letters*.

[92] Espinosa-Neira R, Mejia-Rangel J, Cortes-Reynosa P, Salazar EP (2011). Linoleic acid induces an EMT-like process in mammary epithelial cells MCF10A. *The International Journal of Biochemistry and Cell Biology*.

[93] Kelley NS, Hubbard NE, Erickson KL (2007). Conjugated linoleic acid isomers and cancer. *Journal of Nutrition*.

[94] Amarù DL, Field CJ (2009). Conjugated linoleic acid decreases MCF-7 human breast cancer cell growth and insulin-like growth factor-1 receptor levels. *Lipids*.

[95] Islam MA, Kim YS, Jang WJ (2008). A mixture of trans, trans conjugated linoleic acid induces apoptosis in MCF-7 human breast cancer cells with reciporocal expression of Bax and Bcl-2. *Journal of Agricultural and Food Chemistry*.

[96] Wang LS, Huang YW, Liu S, Yan P, Lin YC (2008). Conjugated linoleic acid induces apoptosis through estrogen receptor alpha in human breast tissue. *BMC Cancer*.

[97] Miglietta A, Bozzo F, Bocca C (2006). Conjugated linoleic acid induces apoptosis in MDA-MB-231 breast cancer cells through ERK/MAPK signalling and mitochondrial pathway. *Cancer Letters*.

[98] Bocca C, Bozzo F, Cannito S, Colombatto S, Miglietta A (2010). CLA reduces breast cancer cell growth and invasion through ER*α* and PI3K/Akt pathways. *Chemico-Biological Interactions*.

[99] Bocca C, Bozzo F, Francica S, Colombatto S, Miglietta A (2007). Involvement of PPAR*γ* and E-cadherin/*β*-catenin pathway in the antiproliferative effect of conjugated linoleic acid in MCF-7 cells. *International Journal of Cancer*.

[100] Huot PSP, Sarkar B, Ma DWL (2010). Conjugated linoleic acid alters caveolae phospholipid fatty acid composition and decreases caveolin-1 expression in MCF-7 breast cancer cells. *Nutrition Research*.

[101] Fay MP, Freedman LS (1997). Meta-analyses of dietary fats and mammary neoplasms in rodent experiments. *Breast Cancer Research and Treatment*.

[102] Ip C (1997). Review of the effects of trans fatty acids, oleic acid, *n* − 3 polyunsaturated fatty acids, and conjugated linoleic acid on mammary carcinogenesis in animals. *American Journal of Clinical Nutrition*.

[103] Rose DP (1997). Effects of dietary fatty acids on breast and prostate cancers: evidence from in vitro experiments and animal studies. *American Journal of Clinical Nutrition*.

[104] Wada M, DeLong CJ, Hong YH (2007). Enzymes and receptors of prostaglandin pathways with arachidonic acid-derived versus eicosapentaenoic acid-derived substrates and products. *The Journal of Biological Chemistry*.

[105] Bagga D, Anders KH, Wang HJ, Glaspy JA (2002). Long-chain *n* − 3-to-*n* − 6 polyunsaturated fatty acid ratios in breast adipose tissue from women with and without breast cancer. *Nutrition and Cancer*.

[106] Murff HJ, Shu XO, Li H (2011). Dietary polyunsaturated fatty acids and breast cancer risk in Chinese women: a prospective cohort study. *International Journal of Cancer*.

[107] Gago-Dominguez M, Yuan JM, Sun CL, Lee HP, Yu MC (2003). Opposing effects of dietary *n* − 3 and *n* − 6 fatty acids on mammary carcinogenesis: the Singapore Chinese health study. *British Journal of Cancer*.

[108] Bernard-Gallon DJ, Vissac-Sabatier C, Antoine-Vincent D (2002). Differential effects of *n* − 3 and *n* − 6 polyunsaturated fatty acids on BRCA1 and BRCA2 gene expression in breast cell lines. *British Journal of Nutrition*.

[109] Bougnoux P, Hajjaji N, Ferrasson MN, Giraudeau B, Couet C, Le Floch O (2009). Improving outcome of chemotherapy of metastatic breast cancer by docosahexaenoic acid: a phase II trial. *British Journal of Cancer*.

[110] Bougnoux P, Hajjaji N, Maheo K, Couet C, Chevalier S (2010). Fatty acids and breast cancer: sensitization to treatments and prevention of metastatic re-growth. *Progress in Lipid Research*.

[111] Altenburg JD, Bieberich AA, Terry C (2011). A synergistic antiproliferation effect of curcumin and docosahexaenoic acid in SK-BR-3 breast cancer cells: unique signaling not explained by the effects of either compound alone. *BMC Cancer*.

[112] Siddiqui RA, Harvey KA, Xu Z, Bammerlin EM, Walker C, Altenburg JD (2011). Docosahexaenoic acid: a natural powerful adjuvant that improves efficacy for anticancer treatment with no adverse effects. *Biofactors*.

